# Are bioplastics the solution to the plastic pollution problem?

**DOI:** 10.1371/journal.pbio.3002045

**Published:** 2023-03-22

**Authors:** Sandra Pascoe Ortiz

**Affiliations:** Universidad del Valle de Atemajac–Guadalajara, Zapopan, Jalisco, Mexico

## Abstract

Bioplastics have been championed as a potential solution to the problem of plastic waste. This Perspective looks at the pros and cons of bioplastics and whether they can live up to their promise.

## Teaser

We live our lives immersed in plastic pollution: a problem that is becoming more acute. Viable alternatives that can reduce plastic pollution are being sought. Could bioplastics be the hoped-for solution to this problem?

In the past decade, the problem of plastic waste has reached unprecedented levels. Macro plastics have moved away from the spotlight and focus has shifted to micro and nano plastics, which are generated by the degradation of non-biodegradable or non-compostable plastics and bioplastics. These materials have been found everywhere, even in the human body, as they are ingested, breathed or absorbed through the skin [1] by animals, humans and plants. Researchers have focused on identifying these materials in human organs and tissues and trying to explain the possible effects that such exposure will have on this and future generations. In fact, modifications have already been found at the mitochondrial level [2] that can lead to disorders in cellular functioning.

Since the 1960s, researchers have been searching for alternatives to petroleum-derived plastics that can replace conventional plastics. These alternatives need to have less impact on the environment, either in their production processes or in that their residues can be treated and incorporated into nature without generating pollution. Among the new materials that have been developed are those known as bioplastics, which generally come from renewable sources (such as plants, animals or microorganisms) and are made from any biological material instead of fossil fuels, but the term can also mean they are biodegradable according to international standards. They are classified either according to their origin or according to their biodegradability: there are those that come from renewable sources and are not biodegradable; those that are produced from fossil sources and are biodegradable; and those that both come from renewable sources and are biodegradable. The first uses of these materials were in medical applications but they are currently used in different products in the agricultural, packaging or textile sectors, to mention a few. Some bioplastics are limited in terms of their mechanical and thermal properties; the main differences to petroleum-derived plastics are their mechanical strength, durability and resistance to high temperatures, as well as their sometimes higher cost [3].

The idea that no bioplastics pollute with their waste is a misconception based on the belief that they all biodegrade or compost but, unfortunately, this is not true. Some bioplastics are neither biodegradable nor compostable, and their residues generate the same pollution problems as those derived from petroleum because they also produce micro and nano plastics during their decomposition. Bioplastics may come from biological material but are chemically the same as petroleum-derived plastic, the only thing that changes is the source from which they are obtained; for example, with Bio-polyethylene terephthalate (Bio-PET), the "Bio" only indicates that its origin is vegetable. This compound is neither biodegradable nor compostable, it is considered a bioplastic only because of its origin. The environmental benefit of this type of material is that, because it comes from a plant, a certain amount of carbon dioxide is captured during the production of its raw material (during the life of those plants). In general terms, the production process of bioplastics compared to petroleum-derived plastics has less of an environmental impact in terms of the balance of greenhouse gas emissions.

It is also important to note that the fact that a bioplastic is biodegradable or compostable does not mean that it can be thrown anywhere and will just disappear. Most biodegradable or compostable bioplastic waste requires processing under controlled conditions to be incorporated back into nature: they must be composted at industrial level. For example, polylactic acid (PLA) takes 80 years in the open air to biodegrade or, if composted industrially, takes days or a few months depending on the conditions of the process [4].

The market for both biodegradable and non-biodegradable bioplastics is growing and these materials have been gaining ground over petroleum-based plastics (although not enough). The main biodegradable bioplastics on the market are polybutylene adipate terephthalate (PBAT), PLA, starch blends, polybutylene succinate (PBS), cellulose films and polyhydroxyalcanoates (PHAs). According to data from European Bioplastic in cooperation with the Nova-Institute from 2021, the most common applications of these materials are in flexible and rigid packaging, consumer goods, textile fibers and in agriculture, and it is projected that by 2026 the production of biodegradable bioplastics will be considerably higher than that of non-biodegradable bioplastics [5].

Bioplastics have several drawbacks. Some the raw materials they use are often also used for food, there is not enough production and their costs are higher than those of conventional plastics. It is often the consumer who has to absorb the price difference and is not in a position to do so, adding another reason why, so far, they have not been able to significantly displace petroleum-based plastics. Bioplastics and biodegradable plastics are part of the solution to the problem of plastic pollution, as they generally have reduced environmental impacts in their production processes and, in some cases, because it is feasible to treat their waste, but they are not the only and absolute solution; the problem of plastic pollution is more complex and is still far from being completely solved. For these materials to reach their full potential, it will be essential to have regulations to regulate their production, certifications in terms of biodegradability and proper education for buyers to choose products that help in the conservation of the environment.

Finally, it should be remembered that pollution is mainly generated by the misuse of materials and poor disposal of their waste. The real problem is the abuse of plastic materials, whether they are biodegradable or not, since they are mainly used in containers, packaging and single-use products, and most of the time they are discarded not because they are useless or their useful life has ended, but because of the convenience of using and throwing away. Certain quantities of plastics can be recycled; however, when they are mixed with other types of waste they become contaminated and when different types of plastic are not adequately separated, this recycling becomes practically impossible. Nevertheless, the recycling of some bioplastics has not yet been trialed, not because it cannot be done, but because of the small quantities of these materials compared to conventional plastics, which makes it practically unaffordable. So, instead of blaming plastic materials for existing environmental pollution, we need to look closely at how we use resources and dispose of waste. No matter how many bioplastics or "environmentally friendly" materials there are, if we do not reduce the production of these types of materials and consequently their waste, there will be no real solutions. We need to be aware of what we consume, support initiatives that promote environmental care and demand the commitment of governments to legislate and enforce laws, as well as encouraging businesses to change their materials and production processes.
